# Antioxidative Effects of Germinated Brown Rice-Derived Extracts on H_2_O_2_-Induced Oxidative Stress in HepG2 Cells

**DOI:** 10.1155/2014/371907

**Published:** 2014-11-06

**Authors:** Nur Diyana Md Zamri, Mustapha Umar Imam, Siti Aisyah Abd Ghafar, Maznah Ismail

**Affiliations:** ^1^Laboratory of Molecular Biomedicine, Institute of Bioscience, Universiti Putra Malaysia, 43400 Serdang, Selangor, Malaysia; ^2^Faculty of Dentistry, Islamic Science University of Malaysia, Level 15, Tower B, Persiaran MPAJ, Jalan Pandan Utama, 55100 Pandan Indah, Kuala Lumpur, Malaysia; ^3^Department of Nutrition and Dietetics, Faculty of Medicine and Health Sciences, Universiti Putra Malaysia, 43400 Serdang, Selangor, Malaysia

## Abstract

The antioxidant properties of germinated brown rice (GBR) are likely mediated by multiple bioactives. To test this hypothesis, HepG2 cells pretreated with GBR extracts, rich in acylated steryl glycoside (ASG), gamma amino butyric acid GABA), phenolics or oryzanol, were incubated with hydrogen peroxide (H_2_O_2_) and their hydroxyl radical (OH^•^) scavenging capacities and thiobarbituric acid-reactive substances (TBARS) generation were evaluated. Results showed that GBR-extracts increased OH^•^ scavenging activities in both cell-free medium and posttreatment culture media, suggesting that the extracts were both direct- and indirect-acting against OH^•^. The levels of TBARS in the culture medium after treatment were also reduced by all the extracts. In addition, H_2_O_2_ produced transcriptional changes in p53, JNK, p38 MAPK, AKT, BAX, and CDK4 that were inclined towards apoptosis, while GBR-extracts showed some transcriptional changes (upregulation of BAX and p53) that suggested an inclination for apoptosis although other changes (upregulation of antioxidant genes, AKT, JNK, and p38 MAPK) suggested that GBR-extracts favored survival of the HepG2 cells. Our findings show that GBR bioactive-rich extracts reduce oxidative stress through improvement in antioxidant capacity, partly mediated through transcriptional regulation of antioxidant and prosurvival genes.

## 1. Introduction

Oxidative stress is implicated in the pathogenesis and progression of a wide variety of chronic diseases including cancers, diabetes, and cardiovascular diseases [1]. Oxidative stress is closely linked to apoptotic cell death, and it is suggested that oxidative stress activates transcriptional mechanisms leading up to apoptosis [2, 3]. The tumor suppressor gene p53, for instance, is activated in response to oxidative stress, which in turn activates multiple pathways including that of the mitogen-activated protein kinases (MAPK). The ultimate outcome of the oxidative stress-induced signals depends largely on the ability of cells to overcome the stimuli for the oxidative stress through negative feedback mechanisms like the activation of cyclooxygenase (COX) II by p53 through various intermediaries belonging to the MAPK and AKT [4–6]. When stress overwhelms the cells, apoptosis sets in, involving various cascades that ultimately cause cell cycle arrest and death.

On the other hand, antioxidants from natural product especially from dietary sources are known to reduce oxidative stress by counteracting the effects of the oxidative-free radicals [1]. Several of these antioxidant-rich dietary sources like germinated brown rice (GBR) are now being studied for their potential role in the management of oxidative stress-related chronic diseases. GBR has been reported to possess high antioxidant capacity, which reduces oxidative stress [7]. Many of the antioxidant effects of GBR are reported to be mediated through transcriptional regulation of antioxidant genes. Usuki et al. (2011) demonstrated that acylated steryl glycoside (ASG) from GBR improved metabolic indices and reduced oxidative stress through induction of insulin-like growth factor 1 (IGF1) [8]. We have also demonstrated that improvement in antioxidant status of type 2 diabetic rats was partly due to transcriptional regulation of antioxidant genes [9].

Molecular targets central to disease progression and management, especially for diet-related chronic diseases, have in recent years received very close attention in view of their central importance in regulating such diet-related diseases [10]. Better understanding of molecular targets involved in GBR's effects may create opportunities to explore the use of GBR or its bioactives as functional food component or nutraceuticals, respectively, for management and/or prevention of diseases as a result of breakdown of pathways in which the molecular targets regulate. Specifically, several molecular targets are involved in oxidative stress regulation and many factors determine how the molecular regulation of oxidative stress proceeds. This makes them important targets in the management of oxidative stress-related conditions. In this regard, dietary factors can be used to regulate these processes by countering oxidative stress [11], the implications of which is that the metabolic perturbations underlying many chronic diseases are targeted and neutralized. As can be recalled, we reported that GBR increases antioxidant status in type 2 diabetic rats [9]. Thus, we extracted the bioactive compounds from GBR (oryzanol, phenolics, gamma aminobutyric acid (GABA), and acylated steryl glycoside (ASG)) and determined their roles in improvement of antioxidant status by GBR and what transcriptional mechanisms they regulate, using HepG2 cells.

## 2. Materials and Methods

### 2.1. Materials

ASG standard was purchased from Matreya (USA), while those of GABA, oryzanol, and phenolics and streptozotocin (STZ), Tris-EDTA (TE) buffer solution, N,O-bis(trimethylsilyl)trifluoroacetamide with 1% trimethylchlorosilane (BSTFA + 1% TMCS), RPMI 1640 medium, fetal bovine serum, and antibiotic were purchased from Sigma-Aldrich. MgCl_2_ and DNA Taq polymerase were purchased from Thermo Fisher Scientific (Pittsburgh, PA), while insulin and other cell cultureware were purchased from Invitrogen and BD Bioscience (NJ, USA), respectively. The total RNA isolation kit was supplied by RBC Bioscience Corp. (Taiwan), and the GenomeLab GeXP Start Kit was purchased from Beckman Coulter Inc. (USA). Hydrogen peroxide (H_2_O_2_) was from Bendosen Laboratory Chemicals (Selangor, Malaysia) and sodium hypochlorite from Dexchem Industries Sdn. Bhd. (Penang, Malaysia). All solvents of analytical grade were purchased from Merck (Darmstadt, Germany).

### 2.2. Germination of Brown Rice

The germination method used was the same as in our publication [12]. Briefly, 100 g of brown rice was weighed and washed 3 times by tap water (2 : 1, water-to-rice ratio) and soaked in 200 mL of 0.1% NaOCl solvent for 30 min. The solvent was drained, and the rice was washed with tap water 5 times. Then, the rice was soaked with 200 mL 0.5% H_2_O_2_ and incubated for 6 h at 37°C. After draining the solution, the rice was incubated for 18 h at 37°C and later dried at 50°C until the moisture content reached 5–8% [12]. GBR was ground into powders with stainless steel grinder (Waring Commercial, Torrington, CT, USA).

### 2.3. Analysis of Bioactives

ASG and extracts rich in GABA, oryzanol, and total phenolics were extracted and analyzed, and their effects on H_2_O_2_-induced oxidative stress in HepG2 cells were determined in this study. The phenolic composition of our 80% methanolic extract has been reported previously [13]. Briefly, 80% methanol was mixed with ground GBR (4 : 1, solvent-to-GBR ratio) and incubated for 2 h in a sonicator (extraction was performed three times), and the resultant supernatants evaporated to dryness (Buchi R-210 rotary evaporator, Rotavap). HPLC analysis (20 *μ*L of sample) was with Agilent G1310A pumps (Agilent, Santa Clara, CA, USA) coupled with a diode array detector set to wavelengths of 280 nm and 320 nm. LUNA C-18 column (5 mm, 250 × 4.6 mm) (Phenomenex, Torrance, CA, USA) was used for separation of phenolics, while the mobile phase was composed of solvent (A) water-acetic acid (94 : 6, v/v, pH 2.27) and solvent (B) acetonitrile, with a gradient as follows: 0–15% B for 40 min, 15–45% B for 40 min, and 45–100% B for 10 min. A flow rate of 0.5 mL/min was used.

GABA extraction was done by adding 4 volumes of cold 70% ethanol and the samples were sonicated for 1 h. The mixture was then centrifuged at 34800 g for 20 min. The resulting pellets were washed further twice with 70% ethanol while the supernatants were concentrated under vacuum and analyzed using HPLC-DAD (Agilent, Santa Clara, CA, USA) after derivatization [14]. Briefly, 5 *μ*L of phenyl isothiocyanate (PITC) was incubated with the samples, which were dissolved in 100 *μ*L of acetonitrile : pyridine : triethylamine : water (10 : 5 : 2 : 3), at room temperature for 5 min. Derivatized samples were then dissolved in 500 *μ*L of buffer A (0.1 M ammonium acetate, pH 6.5), and 20 *μ*L sample was injected into the HPLC system with a C18 reversed-phase column from Alltech (Alltima C18 5U, 250 × 4.6 mm). The following gradient was then used to detect GABA on a diode array detector (wavelength of 254 nm): buffer A (100−0% after 50 min) and buffer B (0.1 M ammonium acetate : acetonitrile : methanol, 44 : 46 : 10, v/v, pH 6.5; 0−100% after 50 min).

Furthermore, oryzanol-rich extract was prepared by adding hexane to GBR (4 : 1, solvent-to-GBR ratio). The same HPLC and detector reported above were used to detect oryzanol at 330 nm, after separation using a C18 column (5 mm ODS, Hypersil Silica), and the mobile phase consisted of acetonitrile : methanol : isopropanol (50 : 45 : 5 v/v) [15].

ASG was analyzed as reported previously [16, 17]. Briefly, total lipid fraction was extracted from GBR using the standard Folch procedure (5 g of rice sample, with 30 mL, then 20 mL of chloroform-methanol, 1 : 1; v/v and 2 : 1; v/v, resp.). The total lipid fraction was then subjected to silica gel column chromatography (15 31.0 cm ID, Iatrobeads, 6RS-8060; Iatron Laboratories, Inc., Tokyo, Japan) and eluted stepwise with the following solvents: (i) 40 mL and 70 mL of chloroform-hexane (1 : 1 and 9 : 1; v/v); and (ii) 80 mL of chloroform-methanol (9 : 1; v/v). As reported previously [16], ASG was analyzed in fraction ii by GC-MS/MS QqQ (Thermo Fischer Scientific, Logan, UT) using a DB-5 column (15 m × 0.25 mm i.d., × 0.25 *μ*m film thickness). Dried extracts and standards were dissolved in pyridine and derivatized with equal volume of BSTFA + 1% TMCS by incubating at 80°C for 90 min. Derivatized samples were then injected into the GCMS in splitless mode and mass spectra (1 scan s^−1^) were acquired from* m/z* 50 to 800 using selected reaction monitoring (SRM). The carrier gas was helium at a flow rate of 1.0 mL/min, while the injector temperature was set at 300°C and the transfer line temperature was 320°C. Initial oven temperature was 120°C and was ramped at 10°C/min until 300°C and held for 5 min.

For HPLC and GCMS analyses and quantification, the compounds were identified by comparing the mass spectra and/or retention times with those of authentic standards.

### 2.4. Treatment of Cells with Extracts and Cell Viability Assay

HepG2 cells were acquired from the American Type Culture Collection (Manassas, VA) and cultured in RPMI 1640 medium supplemented with 10% fetal bovine serum (FBS) and 1% antibiotics (100 U/mL penicillin) in an incubator at 37°C with 5% CO_2_. Cell viability was assessed as reported in our earlier publication [12]. In this experiment, the extracts were dissolved in DMSO (final concentration of <0.1% in all cases) and then used to treat HepG2 cells at nontoxic doses (50 ppm and 100 ppm) of ASG extract (IC50–581 ppm), GABA-rich extract (IC50–785 ppm), phenolic-rich extract (IC50~1000 ppm), and oryzanol-rich extract (IC50~1066 ppm) in a medium that contained 10% FBS and 1% antibiotic. Following 24 h of treatment, 1 mM H_2_O_2_ was added [18] for 4 hours before medium was removed and RNA extracted after washing cells with PBS.

### 2.5. Hydroxyl Radical (OH^•^) Scavenging Activity Determination

The medium from the cell culture was tested for its OH^•^ scavenging potential (*n* = 4) on electron spin resonance (ESR) spectrometer (Jeil FA100; Tokyo, Japan) as reported in our previous publication [9]. Briefly, a mixture of 60 *μ*L of the medium, 40 *μ*L of 0.4 mM DMPO, 37.5 *μ*L of 0.2 mM FeSO_4_, 112.5 *μ*L of 0.2 mM EDTA, and 150 *μ*L of 1 mM H_2_O_2_ were inserted into a quartz cell and measured on the ESR Spectrometer. The parameters used were magnetic field 33.700 ± 5 mT, microwave power 8 MW, modulation frequency 100 KHz, modulation width 0.1 mT, time constant 0.1 s, amplitude 160, and 1 min sweeping time. In a second experiment, the GBR-derived extract was dissolved in cell culture medium and its OH^•^ scavenging potential was tested in comparison to extract-free medium (*n* = 4), using the same conditions above. Peak/marker readings generated from the ESR Spectrometer were used to calculate scavenging activities (%) as follows:

(1) $$\begin{array}{c} \text{Scavenging}\;\text{activity}=\left(\frac{I_{o}-I}{I_{o}}\times 100\%\right), \end{array}$$

where *I*_*o*_ is the control and *I* is the peak/marker value for the different groups.

### 2.6. Thiobarbituric Acid Reactive Species (TBARS) Measurement

As reported by Yang and Koo [19], TBARS measured in cell culture medium is an indicator of oxidative stress. In this study, the medium from the cell culture was used to quantify the amount of TBARS generated by the untreated, H_2_O_2_-treated, and GBR extracts + H_2_O_2_-treated cells (*n* = 4). Briefly, 750 uL of 0.8% thiobarbituric acid was mixed with 750 uL of 20% acetic acid, 100 uL of 8.1% sodium dodecyl sulphate, and 400 uL of the standard 1,1,3,3-tetramethoxypropane or samples in a 2.0 mL tube [19]. Then, the mixture was incubated at 95°C for 1 h and cooled before extraction with 3 mL of n-butanol. Finally, absorbance of the n-butanol extracts was taken on a microplate reader at 532 nm. The malondialdehyde (MDA) equivalents were calculated and expressed as nmol equivalent of MDA using its standard curve equation (*y* = 66.383*x*−2.9131, *R* ^2^ = 0.9679).

### 2.7. RNA Extraction and Gene Expression Study

The transcriptional regulation of antioxidant and apoptosis related genes was also determined in this study, to provide insights into possible mechanisms underlying GBR extracts' protective effects on HepG2 cells (*n* = 4). The primers for the genes of interest and housekeeping genes (Table 1) were designed on NCBI website and purchased from Biosune (Shanghai, China), while the internal control (Kanr) was supplied by Beckman Coulter (USA). RNA was extracted from HepG2 cells at the end of cell culture study using the total RNA isolation kit (RBC Bioscience Corp., Taiwan), according to the manufacturer's instructions. Reverse transcription and PCR were performed according to the GenomeLab GeXP Kit (Beckman Coulter, USA) protocol, in an XP Thermal Cycler (Bioer Technology, Germany). PCR products were finally analyzed on the GeXP genetic analysis system and the results normalized on eXpress Profiler software based on the manufacturer's instructions.

### 2.8. Statistical Analysis

Means of groups were used in the analyses (*n* = 4) and where error bars are shown, they represent standard deviation. One-way analysis of variance (ANOVA) on SPSS 17.0 software (SPSS Inc., Chicago, IL, USA) was used to assess the level of significance between means at *P* < 0.05.

## 3. Results and Discussion

### 3.1. Effects of GBR Extracts on Hydroxyl Radical Scavenging Activity of Culture Medium

**Figure 1:**
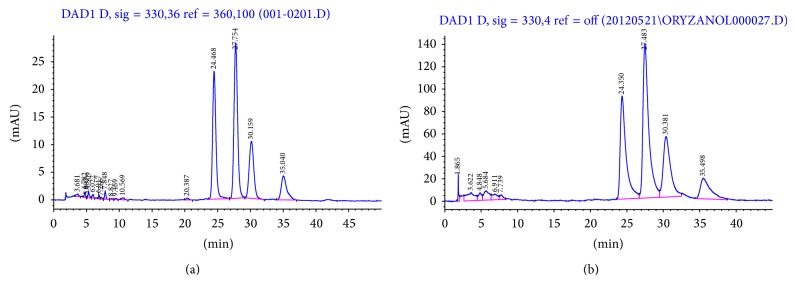
HPLC chromatogram of oryzanol showing (a) oryzanol standard (100 *μ*g/mL) and (b) oryzanol in hexane extract (30 mg/mL) of germinated brown rice. Four isomers of oryzanol were detected corresponding to cycloartenyl ferulate, 24-methylene cycloartanyl ferulate, campestryl ferulate, and mixtures of *β*-sitosteryl ferulate and cycloartanyl ferulate, respectively.

**Figure 2:**
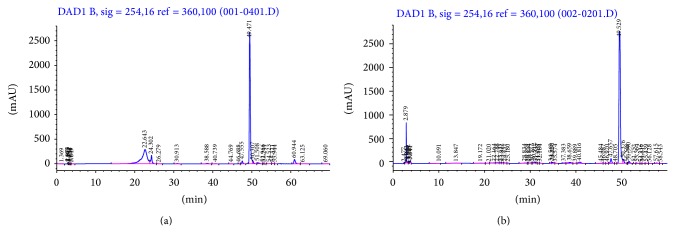
HPLC chromatogram of gamma aminobutyric acid (GABA) showing (a) GABA standard (100 *μ*g/mL) and (b) GABA in ethanolic extract (30 mg/mL) of germinated brown rice. GABA was detected after 49 mins.

**Figure 3:**
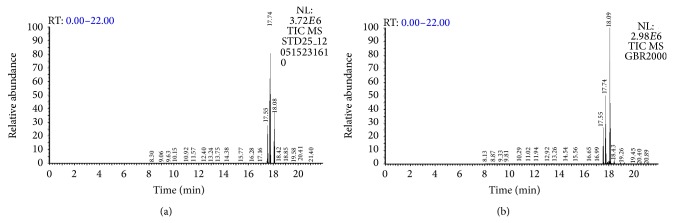
GCMS chromatogram of acylated steryl glycoside (ASG) showing (a) ASG standard (25 *μ*g/mL) and (b) ASG in germinated brown rice extract (2 mg/mL). The 3 peaks detected were campesteryl 3*β*-D-glucopyranoside, stigmasteryl 3*β*-D-glucopyranoside, and sitosteryl 3*β*-D-glucopyranoside, respectively.

**Figure 4:**
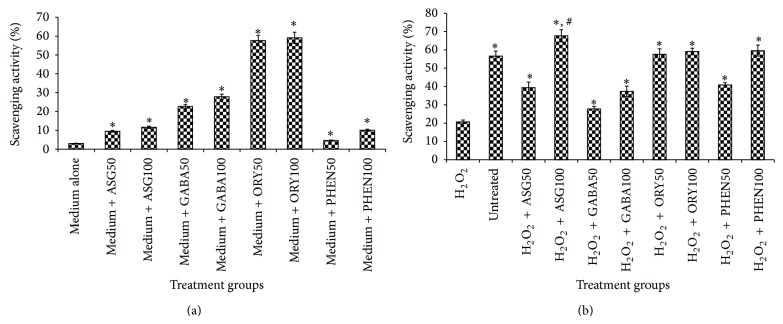
Effects of germinated brown rice extracts on hydroxyl radical scavenging activities (%) of culture media, (a) Cell Free, and (b) after treatment of HepG2 cells (*n* = 4). Treatment groups with 50 or 100 indicate the concentration of bioactive-rich extract used, 50 ppm or 100 ppm, respectively. ASG: acylated steryl glycoside; GABA: gamma amino butyric acid-rich extract; ORY: oryzanol-rich extract; PHEN: phenolic-rich extract. ^*^Significantly higher than control (cell-free medium or H_2_O_2_-treated); ^#^significantly higher than untreated cells.

We have reported earlier that the phenolic composition of the 80% methanolic extract of GBR was found to be 20.5 ± 0.012 mg GAE/g GBR [13]. In addition, the ASG, GABA, and oryzanol composition of GBR extracts were found to be 0.465 ± 0.055 mg/g GBR, 0.36 ± 0.04 mg/g GBR, and 30.38–64.22 mg/100 g GBR, respectively (Figures 1, 2, and 3). In this study, we determined the effects of the bioactive-rich extracts on hydroxyl radical scavenging activity against OH^•^, as shown in Figure 4. In biological systems, OH^•^ can cause oxidative damage to cellular components when present in excess. Antioxidants can scavenge OH^•^ and alleviate its effects directly or indirectly. In this experiment, the abilities of the GBR extracts to scavenge OH^•^ were tested and as shown in Figure 4(a), the presence of different extracts in cell-free culture medium was able to reduce OH^•^ mostly in a dose-dependent manner except for oryzanol-rich extract, which showed the highest scavenging activity in an all-or-none manner. This suggested that the extracts rich in different bioactives possess some antioxidant potential. As shown in Figure 4(b), however, treatment of HepG2 cells with the GBR extracts in the presence of H_2_O_2_ markedly increased the OH^•^ scavenging capacity of the medium except for oryzanol-rich extract. This suggested that the OH^•^ scavenging capacity in the medium due to oryzanol-treated cells was largely contributed by the antioxidant potentials of the extract in the medium or it likely induced changes in the cells that were not detectable in the culture medium. In the case of all other extracts, the results suggested that, in addition to the antioxidant potentials of the bioactives, stimulation of antioxidant defence systems in the cells could have produced more antioxidants that were released into the medium to scavenge more OH^•^. This data is in agreement with previous reports on the mechanisms of action (direct and indirect) of antioxidants [20]. In addition, GBR has been reported to have high antioxidant potentials and the present results show that the antioxidant effects of GBR may likely be contributed by multiple bioactives through food synergy. This is in keeping with our previous findings on the contributions of GBR bioactives to its functional effects [12, 21].

### 3.2. Effects of GBR Extracts on TBARS Generation in Culture Medium

**Figure 5:**
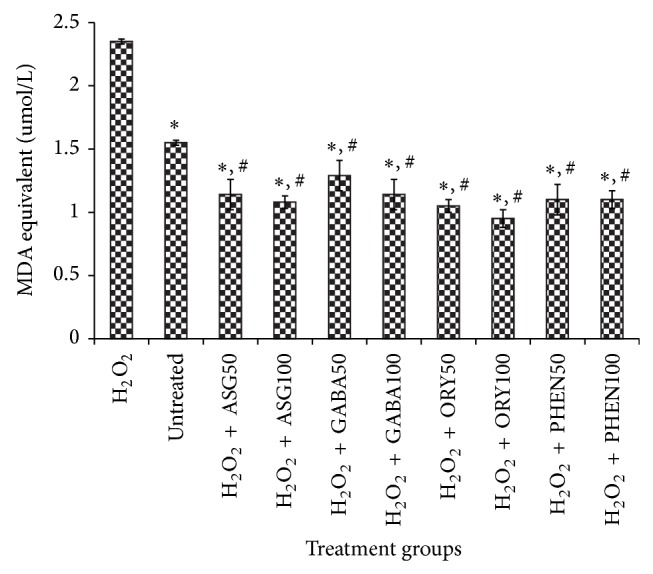
Effects of germinated brown rice extracts on thiobarbituric acid reactive species (TBARS) generation in culture media after treatment of HepG2 cells (*n* = 4). ^*^Significantly lower than H_2_O_2_-treated; ^#^significantly lower than untreated cells. Treatment groups are the same as in Figure 4.

Oxidative stress results in increased lipid peroxidation, generated through free radical damage of cell membrane lipids. To determine lipid peroxidation, TBARS like MDA are mostly measured. As shown in Figure 5, all extracts from GBR were able to reduce the MDA levels in culture medium indicating that oxidative stress was alleviated in the HepG2 cells. In fact, MDA levels in treated cells in the presence of H_2_O_2_ were lower than in untreated cells suggesting that the bioactives could reduce oxidative stress lower than basal levels. The reduction in MDA levels in the culture medium due to the extracts corroborates earlier findings that GBR reduced oxidative stress [7]. In addition, this study shows that the alleviation of oxidative stress by GBR may be contributed by more than one factor.

### 3.3. Regulation of JNK, p53, p38 MAPK, AKT, and Antioxidant Genes in HepG2 Cells

**Figure 6:**
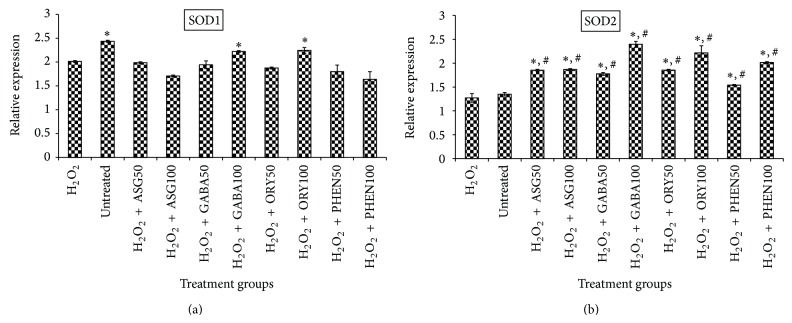
Effects of germinated brown rice extracts on mRNA levels of superoxide dismutase (SOD) genes in HepG2 cells (*n* = 4). ^*^Significantly higher than H_2_O_2_-treated; ^#^significantly higher than untreated cells. Treatment groups are the same as in Figure 4.

**Figure 7:**
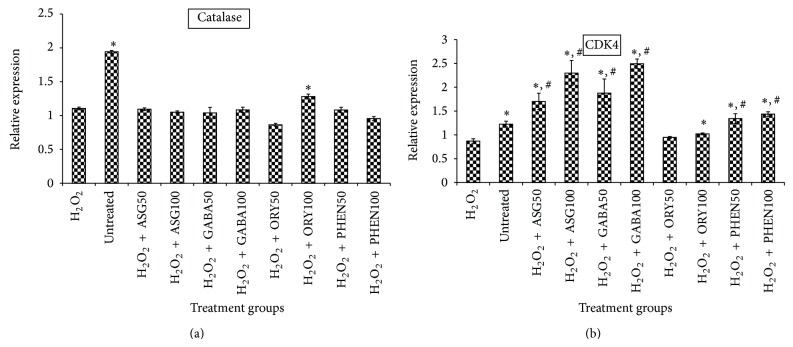
Effects of germinated brown rice extracts on mRNA levels of Catalase and cyclin-dependent kinase (CDK) 4 genes in HepG2 cells (*n* = 4). ^*^Significantly higher than H_2_O_2_-treated; ^#^significantly higher than untreated cells. Treatment groups are the same as in Figure 4.

As would be recalled, the results of the OH^•^ scavenging activity in Figure 4 indicated that GBR extracts produced their antioxidative effects both directly and indirectly. To determine the likely transcriptional mechanisms involved in their indirect effects, antioxidant, proapoptotic, and prosurvival genes were studied.  Figure 6 shows the mRNA levels of superoxide dismutase (SOD) 1 and SOD 2 in HepG2 cells. The results show that all extracts increased mRNA levels of SOD 2 in a dose-dependent manner in the presence of H_2_O_2_, but only oryzanol- and GABA-rich extracts increased those of SOD 1. In the case of Catalase (Figure 7), however, only oryzanol-rich extract was able to upregulate its expression. The oryzanol-rich extract upregulated the expression of antioxidant genes although it did not produce detectable increase in OH^•^ scavenging activity in posttreatment culture medium, compared to cell-free medium levels, indicating that it induced protective changes in the cells, thereby protecting them from damage by the radicals.

Oxidative stress and apoptotic cell death are known to have close links. In fact, apoptosis is often the end result of oxidative damage on cells. In this study we hypothesized that GBR extracts may regulate some processes leading up to apoptotic cell death as part of their antioxidative effects [22]. CDK4 is a cell cycle regulator, and its expression is essential for continuous cell survival. When CDK4 is inhibited, the end result is cell cycle arrest and death, through apoptosis. CDK4 expression in the current study in H_2_O_2_-treated cells was significantly lower than in untreated cells (Figure 7), suggesting that H_2_O_2_ tended to cause cell cycle arrest. However, in the presence of the GBR extracts, there was upregulation of CDK4 indicating that the bioactive-rich extracts likely induced survival factors that favored cell survival instead of death even in the presence of H_2_O_2_.

**Figure 8:**
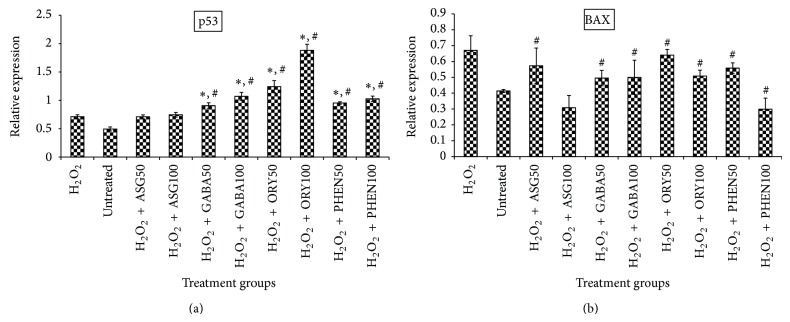
Effects of germinated brown rice extracts on mRNA levels of tumor suppressor gene p53 and Bcl-2-associated X (BAX) genes in HepG2 cells (*n* = 4). ^*^Significantly higher than H_2_O_2_-treated; ^#^significantly higher than untreated cells. Treatment groups are the same as in Figure 4.

**Figure 9:**
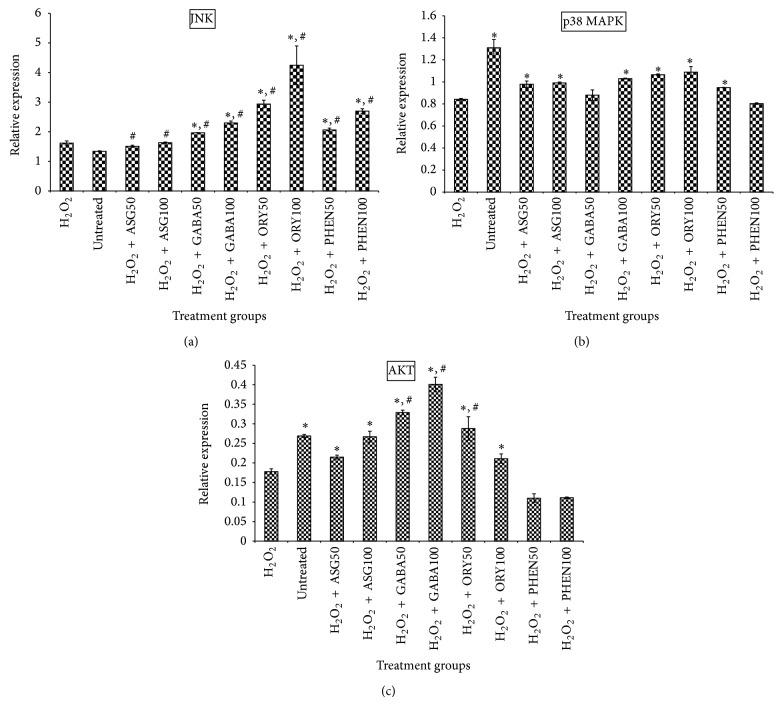
Effects of germinated brown rice extracts on mRNA levels of c-Jun N-terminal kinase (JNK), p38 mitogen-activated protein kinase (p38 MAPK), and v-akt murine thymoma viral oncogene homolog 1 (AKT) genes in HepG2 cells (*n* = 4). ^*^Significantly higher than H_2_O_2_-treated; ^#^significantly higher than untreated cells. Treatment groups are the same as in Figure 4.

Furthermore, p53 expression in the H_2_O_2_-treated cells was upregulated. The tumor suppressor gene p53 is generally activated by oxidative stress including H_2_O_2_ and leads to activation of multiple pathways, some of which may lead to cell destruction. p53 induction by H_2_O_2_ may have resulted in activation of BAX, a proapoptotic factor, in the present study (Figure 8) further confirming that H_2_O_2_ tended to push cells into apoptotic death. Interestingly, all extracts except the ASG-rich fraction upregulated p53 and BAX in this study despite showing signs of improved antioxidant status (Figure 4) and reduced oxidative stress (Figure 5). It is known, however, that response of cells generally determines whether survival or death ensues in the presence of oxidative stress. For instance, COX-II counteracts p53-induced processes leading up to oxidative stress [4, 6]. Also, complex processes closely linked to p53 involving MAPKs like JNK and p38 MAPK [23] may, in response to oxidative insults and activation of p53, protect cells from harmful effects through activation of other pathways that counter the effects of oxidative stress [23, 24]. Moreover, in this study, there was significant upregulation of JNK and p38 MAPK by GBR extracts in comparison to cells treated with H_2_O_2_ alone (Figure 9), suggesting that despite the presence of H_2_O_2_, survival factors were likely induced by the bioactive-rich extracts and tended to push the cells into survival mode. This conforms to the results of the CDK4 gene expression, which showed that the extracts were maintaining the cells in a survival mode in cell cycle. Furthermore, upregulation of AKT is thought to prevent the activation of death signals, also through activation of COX-II [5]. COX-II is known to have a negative feedback effect on p53, thus regulating its proapoptotic tendencies [4, 6]. The results of AKT expression in this study showed that H_2_O_2_ downregulated its expression, which would likely lead to apoptosis, while treatment with GBR extracts, except phenolic-rich extract, upregulated AKT.

By and large, the findings from this study suggest that multiple transcriptional factors may be involved in the protection of the HepG2 cell against H_2_O_2_-induced oxidative stress in the presence of GBR extracts. While GBR bioactive-rich extracts upregulated p53 and BAX similar to what H_2_O_2_ produced, transcriptional activation of other prosurvival factors (JNK, p38 MAPK, and AKT) and antioxidants (Catalase and SOD) likely protected HepG2 cells from the harmful effects of H_2_O_2_-induced oxidative stress. Moreover, in this study all extracts upregulated SOD 2, an important process that blocks apoptosis [11]. As expected, this study showed that, in the presence of H_2_O_2_, oxidative stress would activate transcriptional factors and signal transducers like p53 and MAPKs [23], which may eventually activate cell destruction. In addition, the upregulation of BAX suggested that H_2_O_2_-induced apoptosis in the cells [22]. However, in the presence of the GBR extracts, there were indications that the cells' antioxidant systems were upregulated to protect the cells and reduce oxidative stress (TBARS). Thus, the cells tended to go into survival mode (CDK4 upregulation), likely induced by multiple mechanisms involving transcriptional factors and signal transducers like JNK, p38 MAPK, and AKT. These findings suggest that, in addition to endogenous antioxidant systems induced due to oxidative stress, GBR extracts are able to, directly or indirectly, assist cells to alleviate oxidative stress [20].

Dietary antioxidants have been widely reported to induce antioxidants in biological systems, and this is thought to be the basis for the disease-preventing and health promoting effects of many antioxidant-rich foods like GBR [1]. In this study, we demonstrated that antioxidant effects of GBR are likely mediated through the ability of its multiple bioactive compounds to improve antioxidant status in biological systems thereby reducing oxidative stress. We also demonstrated that transcriptional regulation of antioxidant and prosurvival genes by the GBR extracts likely plays key roles in protecting cells from oxidative stress. These findings are worth studying further in in vivo systems.

## 4. Conclusions

As documented in our earlier study, GBR improved OH^•^ scavenging activity in type 2 diabetic rats. In addition, our current results show that the different bioactives from GBR may contribute towards the overall effect of GBR towards improving OH^•^ scavenging activity. In this study, GBR extracts were able to counter the oxidative damage induced by H_2_O_2_ on HepG2 cells as evidenced by improved OH^•^ scavenging activity of posttreatment medium and reduced TBARS levels, partly through transcriptional regulation of antioxidant (Catalase and SOD) and prosurvival genes (JNK, AKT, and p38 MAPK). The induction of antioxidative changes (reduction in TBARS level and upregulation of antioxidant, JNK, p38 MAPK, and AKT genes) in posttreatment culture medium and cells despite lack of improvement of OH^•^ scavenging activity by oryzanol-rich extract in posttreatment medium compared to the cell-free medium suggested that it also protected the cells from H_2_O_2_-induced oxidative damage like other extracts. The overall effects of the extracts, therefore, indicate that they may directly and/or indirectly relieve oxidative stress. Taken together, our data suggest that different bioactives likely contribute towards the antioxidative effects of GBR, through food synergy, and are worth studying further.

## Figures and Tables

**Table 1:** Gene name and sequences of primers used in the multiplex panel.

Gene name	Forward primer sequence	Reverse primer sequence
SOD 1	AGGTGACACTATAGAATATCATCAATTTCGAGCAGAAGG	GTACGACTCACTATAGGGATGCTTTTTCATGGACCACC
SOD 2	AGGTGACACTATAGAATACATCAAACGTGACTTTGGTTC	GTACGACTCACTATAGGGACTCAGCATAACGATCGTGGTT
Catalase	AGGTGACACTATAGAATAGAAGTGCGGAGATTCAACACT	GTACGACTCACTATAGGGAACACGGATGAACGCTAAGCT
AKT	AGGTGACACTATAGAATAGAGGAGATGGACTTCCGGTC	GTACGACTCACTATAGGGAAGGATCTTCATGGCGTAGTAGC
P53	AGGTGACACTATAGAATAGGGGAGCAGGGCTCA	GTACGACTCACTATAGGGAAAAATGGCAGGGGAGGG
P38 MAPK	AGGTGACACTATAGAATATTCAGTCTTTGACTCAGATGCC	GTACGACTCACTATAGGGAGTCAGGCTTTTCCACTCATCT
JNK	AGGTGACACTATAGAATACAGAAGCTCCACCACCAAAGAT	GTACGACTCACTATAGGGAGCCATTGATCACTGCTGCAC
BAX	AGGTGACACTATAGAATACCCTTTTGCTTCAGGGTTTC	GTACGACTCACTATAGGGACAAAGTAGAAAAGGGCGACAA
CDK4	AGGTGACACTATAGAATAGTCAAGCTGGCTGACTTTGG	GTACGACTCACTATAGGGATCGACGAAACATCTCTGCAA
Peptidylprolyl isomerase A^∗#^	AGGTGACACTATAGAATACACACGGCTCACATTGCAT	GTACGACTCACTATAGGGACACGAACAGCAAAGCGA
Beta actin^*^	AGGTGACACTATAGAATAGATCATTGCTCCTCCTGAGC	GTACGACTCACTATAGGGAAAAGCCATGCCAATCTCATC
Glyceraldehyde-3-phosphate dehydrogenase^*^	AGGTGACACTATAGAATAAAGGTGAAGGTCGGAGTCAA	GTACGACTCACTATAGGGAGATCTCGCTCCTGGAAGATG

^*^Housekeeping genes. ^#^Used for normalization. Reverse transcription (RT) and PCR were done according to manufacturer's instructions; RT reaction was at 48°C for 1 min; 37°C for 5 min; 42°C for 60 min; 95°C for 5 min; then hold at 4°C, while PCR was as follows: initial denaturation at 95°C for 10 min, followed by two-step cycles of 94°C for 30 sec and 55°C for 30 sec, ending in a single extension cycle of 68°C for 1 min. AKT: v-akt murine thymoma viral oncogene homolog 1; BAX: Bcl-2-associated X; CDK4: cyclin-dependent kinase 4; JNK: c-Jun N-terminal kinases; p38 MAPK: p38 mitogen-activated protein kinase; p53: tumor suppressor p53; SOD: superoxide dismutase.

## Abbreviations

ASG: Acylated steryl glycoside
AKT: v-akt murine thymoma viral oncogene
BAX: Bcl-2-associated X
CDK4: Cyclin-dependent kinase 4
COX: Cyclooxygenase
DMPO: 5,5-Dimethyl-pyrroline N-oxide
EDTA: Ethylenediaminetetraacetic acid
ESR: Electron spin resonance
FESO_4_: Ferrous sulphate
GABA: Gamma aminobutyric acid
GBR: Germinated brown rice
H_2_O_2_: Hydrogen peroxide
IGF1: Insulin-like growth factor 1
JNK: c-Jun N-terminal kinase
MDA: Malondialdehyde
OH^•^: Hydroxyl radical
p38 MAPK: p38 mitogen-activated protein kinases
p53: Tumor suppressor gene 53
SOD: Superoxide dismutase
TBARS: Thiobarbituric acid reactive substances.
